# Embedded Physical Intelligence in Liquid Crystalline Polymer Actuators and Robots

**DOI:** 10.1002/adma.202312313

**Published:** 2024-03-27

**Authors:** Wei Feng, Qiguang He, Li Zhang

**Affiliations:** ^1^ Department of Mechanical and Automation Engineering The Chinese University of Hong Kong Hong Kong China

**Keywords:** liquid crystals, physical intelligence, robotics

## Abstract

Responsive materials possess the inherent capacity to autonomously sense and respond to various external stimuli, demonstrating physical intelligence. Among the diverse array of responsive materials, liquid crystalline polymers (LCPs) stand out for their remarkable reversible stimuli‐responsive shape‐morphing properties and their potential for creating soft robots. While numerous reviews have extensively detailed the progress in developing LCP‐based actuators and robots, there exists a need for comprehensive summaries that elucidate the underlying principles governing actuation and how physical intelligence is embedded within these systems. This review provides a comprehensive overview of recent advancements in developing actuators and robots endowed with physical intelligence using LCPs. This review is structured around the stimulus conditions and categorizes the studies involving responsive LCPs based on the fundamental control and stimulation logic and approach. Specifically, three main categories are examined: systems that respond to changing stimuli, those operating under constant stimuli, and those equip with learning and logic control capabilities. Furthermore, the persisting challenges that need to be addressed are outlined and discuss the future avenues of research in this dynamic field.

## Graphical abstract

Liquid crystal polymers have garnered substantial interest in the pursuit of intelligent robots, thanks to their capacity for reversible shape transformation and the vast potential for manifesting physical intelligence. In this review, a focused summary of the stimulation methodologies employed from various perspectives is provided and discusses current research trends in robotics to imbue physical intelligence into LC polymer systems.

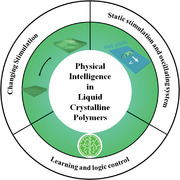

## 1 Introduction

Intelligence is a pervasive phenomenon in the natural world, manifesting itself across various life forms. Beginning with the unicellular organisms, for example, Paramecium shows the capacity to perceive environmental cues and subsequently decide whether to disperse away from or congregate around these stimuli.^[^ ^1^ ^]^ Moving up the hierarchy of cognitive abilities, humans, the most intellectually advanced species, possess the capability to not only recognize and respond to stimuli but also to fashion tools for addressing the challenges presented by these stimuli. Physical intelligence is a promising approach to developing autonomous robots especially miniature robots. Distinguishing itself from artificial or virtual intelligence, physical intelligence pertains to the innate responsiveness of objects to external stimuli, which arises from the intrinsic properties of the materials involved rather than relying on external circuits and CPU‐based algorithms for decision‐making.^[^ ^2^ ^]^ To achieve physical intelligence, the material itself must exhibit a capacity for stimulus‐responsive behaviors and possess the ability to make decisions based on its inherent properties. Among the array of responsive materials with the capacity for reversible shape changes in response to external stimuli, liquid crystalline polymers (LCPs) have garnered substantial attention within scientific research. This responsive characteristic makes them compelling subjects for extensive investigation and study.^[^ ^3^, ^4^, ^5^, ^6^, ^7^, ^8^ ^]^

LCPs represent a class of polymers characterized by the alignment of mesogenic units. Currently, most liquid crystals used for actuators are calamitic liquid crystals. Other types of liquid crystals, such as discotic liquid crystals and bent‐core liquid crystals, also play important roles in applications such as charge transfer, energy storage, and light modulation.^[^ ^9^, ^10^, ^11^ ^]^ More comprehensive summary of these liquid crystals can be found in previous reviews.^[^ ^9^, ^12^, ^13^, ^14^ ^]^ This review will mainly focus on calamitic liquid crystals. Geometric attributes of these polymers, owing to the rod‐like nature of calamitic liquid crystals, impart both geometric and mechanical anisotropy upon them. Responsive LCPs are typically categorized into two types: liquid crystal networks (LCNs) and liquid crystal elastomers (LCEs). LCNs were initially pioneered by Broer and coworkers, who achieved their formation by directly photopolymerizing acrylated small‐molecular liquid crystal mesogens into a rigid polymer network.^[^ ^15^, ^16^ ^]^ LCEs are often produced by mechanically stretching partially polymerized liquid crystal films to orient the liquid crystal mesogens,^[^ ^17^, ^18^ ^]^ followed by a subsequent polymerization step.^[^ ^4^, ^5^, ^19^, ^20^ ^]^ There is no strict definition and obvious classification of LCNs and LCEs. But typically, LCNs refer to the polymeric systems directly (photo)polymerized from low‐molecular‐weight reactive mesogens,^[^ ^21^, ^22^ ^]^ usually with high crosslinking density, relatively high glass transition temperature, rigid and low stretchability (<≈20%); while LCEs usually refer to stretchable LCPs with relatively high stretchability and elasticity.^[^ ^17^, ^18^ ^]^ Various methods can be used to align LC mesogens during fabrication, and representative alignment methods for the fabrication of LCPs are listed in **Table** **1**.

**Table 1:** Representative alignment methods to fabricate LCPs actuators/robots.

Alignment method	Mechanism/principle	Alignment direction	Refs.
Microgrooves	Manual rubbing to generate microgrooves on the alignment layer (polyimide or PVA).	Uniaxial on the same substrate. Different substrates can be combined to generate an orthogonal alignment direction.	[6, 23]
	Micro‐rubbing on the alignment layer using a small metallic sphere.	Different planar directions to generate multiple domains	[24, 25]
	Microchannel fabricated via MEMS.	Use microchannels (≈1 µm wide) to align liquid crystal mesogens in planar directions	[8, 19, 26]
Photoalignment	Use polarized light to orient or photochemically crosslink photoreactive reagents to produce alignment force for the liquid crystal mesogens.	Planar alignment. Photomasks and multiple light exposure steps are used to generate multiple domains.	[5, 27, 28, 29]
Surface anchoring	Use alignment layers with low surface energy (usually alkylated polyimide) or textured surfaces to induce vertical alignment.	Vertical alignment (homeotropic).	[30, 31]
Self‐assembly	By combining surface anchoring force, use chiral dopants or immiscible substances to introduce self‐assembly of liquid crystal mesogens.	Multiple domains.	[32, 33, 34, 35]
Magnetic field	Magnetic field orients the liquid crystal mesogens.	The alignment direction is along the magnetic field direction, independent of the geometric principal axis of LCE actuators.	[4, 36, 37, 38, 39, 40, 41]
Electric field	Electric field orients the dielectric liquid crystal mesogens.	Liquid crystals orient along or perpendicular to the electric field direction, depending on the dielectric anisotropy of liquid crystals.	[42, 43, 44, 45]
Mechanical stretch	Stretch partially crosslinked LCE and LC mesogens orient along the stretch direction, with a following polymerization to fix the orientation.	Uniaxial along the stretch direction.	[17, 18]
Shear stress	Use the ink direct printing method to direct the alignment of LCEs; or along the shear direction.	Along the printing path or shear direction.	[46, 47, 48, 49]

The orientation of the long molecular axes of liquid crystal mesogens is conventionally referred to as the “director” of the liquid crystals. Its degree of alignment is typically quantified by the scalar order parameter *S*. The actuation principle is based on the anisotropic deformation of aligned LCPs. The decrease in order parameter results in anisotropic deformation of the liquid crystal, where a uniaxially aligned LCP contracts along the director and expands in the orthogonal direction.^[^ ^50^ ^]^ Hybrid alignments (e.g., slay or chiral nematic) are usually adopted, and an alignment gradient is engineered along the LCN film thickness direction. Upon actuation and decrease of the order parameter, the anisotropic deformation of LCNs on different sides generates cooperative strain, inducing the deformation of the LCN actuator. The thickness of LCN is limited up to ≈100 µm due to degraded anchoring force along the thickness from the alignment layer. In contrast, LCEs can be fabricated with the thickness of up to several millimeters. The deformation in both LCNs and LCEs can be induced by various stimuli, including electric fields,^[^ ^32^, ^51^, ^52^ ^]^ light,^[^ ^3^, ^53^, ^54^, ^55^ ^]^ and heat via photo‐/electro‐thermal effects.^[^ ^30^, ^56^, ^57^, ^58^ ^]^ Different from the one‐way shape deformation of shape memory polymers that only exhibit one cycle of stimuli‐responsive shape deformation,^[^ ^59^ ^]^ LCPs exhibit reversible shape change upon application/removal of stimuli, making them suitable for the development of robots and actuators with repeatable actuation.

The deformation behavior or mode is endowed to the LCPs via encoding the spatial alignment (gradient), either in the direction along,^[^ ^5^, ^8^, ^32^, ^51^, ^60^ ^]^ perpendicular^[^ ^3^, ^6^ ^]^ or independent^[^ ^4^, ^36^ ^]^ of the geometrical principal axis of LC polymer‐based actuators/robots. The responsive behavior of these actuators/robots in the actuation process is free of digital algorithms that are commonly used in artificial intelligence, and therefore is usually referred to as “physical intelligence”.^[^ ^2^ ^]^

In this review, we will summarize recent developments and present representative examples, classifying them into three autonomy levels of developed actuators or devices: I. alternating stimuli, II. static stimuli and oscillating response, III. adaptive physical intelligence and associated learning. Here we primarily focus on exploring embodied intelligence within LCPs, with a deliberate choice not to delve extensively into the myriad aspects of LCPs, as comprehensively covered in previous reviews.^[^ ^9^, ^61^, ^62^ ^]^

## 2 Actuation using Changing Stimuli

### 2.1 Monotonic Response: Responsive LCPs Using Manually Changing Stimuli to Actuate LCPs into Discrete States

**Figure 1:**
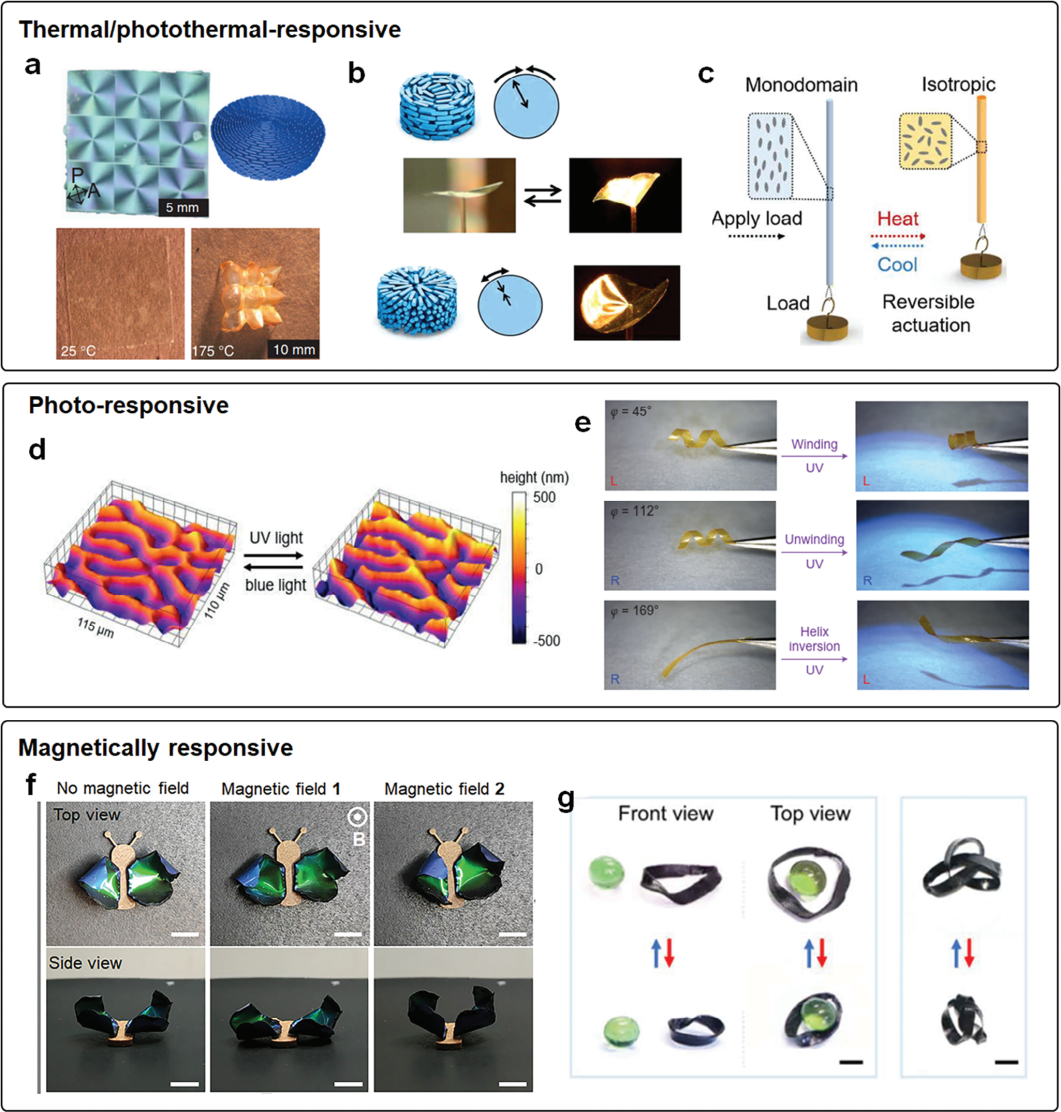
Thermal‐/photo‐/magnetically responsive LCP actuators. a) Thermal‐responsive LCE sheets with engineered molecular alignment turn flat to corrugated upon heating. Reproduced with permission.^[^ ^5^ ^]^ Copyright 2015, The American Association for the Advancement of Science (AAAS). b) Photo‐thermal responsive LCN films curl in response to the photothermal effect, and the engineered molecular alignment determines the shape change during actuation. Reproduced with permission.^[^ ^60^ ^]^ Copyright 2012, Wiley‐VCH. c) Thermal responsive deformation of uniaxially aligned LCE fiber. Reproduced with permission.^[^ ^7^ ^]^ Copyright 2021, AAAS. d) Light‐induced invertible surface topography of the LCN polymer coating encoded with dichroic dye. Reproduced with permission under the CC BY 4.0 license.^[^ ^73^ ^]^ Copyright 2021, The Authors, published by Wiley‐VCH. e) Light‐induced shape change of the free‐standing LCN polymer strips. Reproduced with permission.^[^ ^6^ ^]^ Copyright 2014, Springer Nature. f) Magnetically actuated LCN/elastomer composites. Reproduced with permission.^[^ ^67^ ^]^ Copyright 2023, Wiley‐VCH. g) Shape change of the magnetic LCEs in response to alternating magnetic fields. Reproduced with permission under the CC BY 4.0 license.^[^ ^75^ ^]^ Copyright 2022, The Authors, published by AAAS.

The direct actuation method involves the application of stimuli to induce an actuated state and subsequently removing the stimulation by deactivating it. This process results in two distinct states: the actuated state and the unactuated state. One of the most straightforward approaches involves modifying environmental factors, such as through heating,^[^ ^5^, ^8^, ^63^, ^64^, ^65^ ^]^ the photothermal effect^[^ ^60^, ^66^ ^]^ or humidity.^[^ ^23^, ^67^, ^68^ ^]^ Upon applying external stimuli, LCPs transit from an ordered state (e.g., nematic, smectic, cholesteric, or blue phase) to a disordered state, resulting in a significant shape change.^[^ ^5^, ^30^, ^67^, ^69^, ^70^, ^71^, ^72^ ^]^ LCPs can fully recover to their initial shapes once the stimuli are removed, exhibiting exceptional reversibility. Various forms of LCP actuators and robots have been developed, including free‐standing films, coatings, fibers, and colloidal particles.^[^ ^5^, ^7^, ^60^, ^73^, ^74^ ^]^ Ware et al. demonstrated the reversible transformation of voxelated LCEs between flat and corrugated shapes by imprinting a +1 topological defect array into the LCEs (**Figure** **1**a).^[^ ^5^ ^]^ Haan et al. demonstrated the deformation of LCN films with azimuthal and radial alignments when subjected to photothermal actuation (Figure 1b).^[^ ^60^ ^]^ He et al. reported the thermal‐assisted actuation of LCE in the form of fibers (Figure 1c).^[^ ^7^ ^]^

Except for heating, light can also be used to actuate the deformation of LCPs in the means of photochemical/photomechanical reaction of azobenzene chromophores. For example, Liu et al. harnessed the photomechanical effect of azobenzene to trigger the topographical deformation of chiral nematic LCN coatings with alternating homeotropic/planar alignments, creating a “fingerprint” configuration.^[^ ^76^ ^]^ The initial relative height of homeotropic and planar domains was further regulated via the strategy using dichroic compounds during polymerization, thus leading to different deformation modes‐increased roughness or inverted surface topography‐upon actuation.^[^ ^35^ ^]^ With reversible topographical inversion, this coating can be triggered by UV light and returned to its initial state through exposure to visible light (Figure 1d). Notably, it was also shown to alter surface adhesion underwater, where the polymer coatings were attached to rigid glass substrates.^[^ ^73^ ^]^ The on‐off control of light was also demonstrated to induce shape changes in free‐standing LCPs.^[^ ^6^, ^53^, ^77^, ^78^, ^79^, ^80^, ^81^ ^]^ For instance, twisted nematic LCNs could switch between helical structures using light (Figure 1e).^[^ ^6^ ^]^ Additionally, alternative methods, such as magnetic fields, were employed to deform the LCP actuators. Magnetic particles were either blended with other elastomeric matrices (e.g., Ecoflex) to form a multi‐layer structure with LCPs (Figure 1f) or directly incorporated into LCPs to make them sensitive to magnetic fields (Figure 1g).^[^ ^67^, ^75^, ^82^ ^]^ The magnetic particles integrated into the actuators can be either hard magnetic to be actuated via magnetic torque or soft magnetic to be actuated via alternating magnetic field‐induced heating.

**Figure 2:**
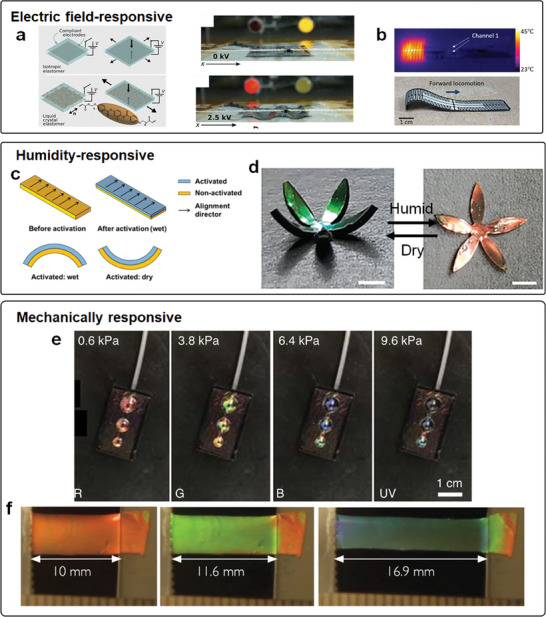
Electric field‐/humidity‐/mechanically responsive LCPs. a) Electrically driven topographical deformation of dielectric LCE sheet. Reproduced with permission under the CC BY 4.0 license.^[^ ^30^, ^83^ ^]^ Copyright 2019, The Authors, published by AAAS. b) Electrically driven soft crawling LCE robot with distributed electrothermal actuation. Reproduced with permission under the CC BY 4.0 license.^[^ ^58^ ^]^ Copyright 2023, The Authors, published by AAAS. c) Humidity‐responsive LCN actuator of uniaxial alignment with partially activated areas. Reproduced with permission.^[^ ^23^ ^]^ Copyright 2014, American Chemical Society (ACS). d) Humidity‐responsive synergistic color and shape change of cholesteric liquid crystal actuators. Reproduced with permission under the CC BY 4.0 license.^[^ ^67^ ^]^ Copyright 2023, The Authors, published by Wiley‐VCH. e) Inflating chiral nematic liquid crystalline elastomer with controlled structural color via pneumatic approach. Reproduced with permission.^[^ ^87^ ^]^ Copyright 2022, Springer Nature. f) Tunable structural color of LCE through mechanical stretch. Reproduced with permission under the CC BY 4.0 license.^[^ ^86^ ^]^ Copyright 2020, The Authors, published by Wiley‐VCH.

Other stimuli can also be used to actuate the deformation of LCPs. Electric field as a remotely controlled stimulus shows the promise of further integration with electronics, therefore attracting intensive interest.^[^ ^32^, ^51^, ^56^, ^57^, ^83^, ^84^ ^]^ The underlying actuation mechanism of these actuators is either based on the dielectric anisotropy of liquid crystals (**Figure** **2**a) or solely based on the electrothermal effect (Figure 2b).^[^ ^32^, ^57^, ^83^ ^]^ The strategy based on LC's dielectric anisotropy usually requires high driving voltage and command the development of new LC materials or new actuator designs despite recent impressive progress.^[^ ^52^, ^85^ ^]^ In contrast, approaches based on electrothermal effect/joule heating often require relatively low voltage, but it will be greatly influenced by heat transfer and corresponding application may be hindered in the medium with high thermal conductivity/capacity, for example, water. The deformation of liquid crystal actuators can also be triggered by humidity. This is achieved by using hygroscopic liquid crystal mesogens, for example, hydroxyl carboxylate groups, to make the LCN swellable in the atmosphere with high humidity (Figure 2c,d).^[^ ^23^, ^67^ ^]^ Besides, some LCPs are also responsive to direct mechanical strain including stretch and compression, behaving as the change in the structural color (Figure 2e,f).^[^ ^86^, ^87^, ^88^, ^89^, ^90^ ^]^ The change of structural color has been demonstrated to be useful in information encryption and storage.

### 2.2 Continuous Oscillation/Actuation of the LCPs using Engineered Stimuli

**Figure 3:**
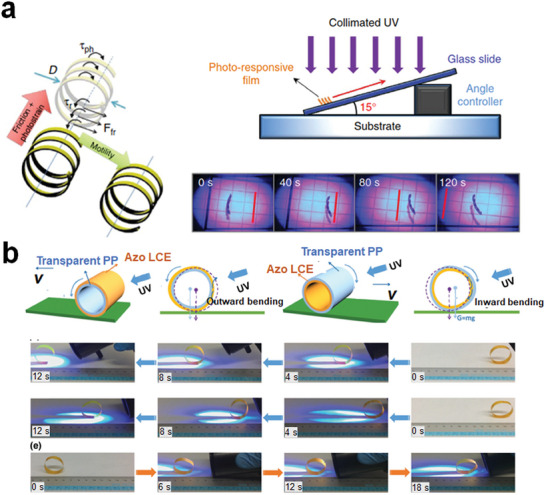
Continuous actuation under changing stimuli. a) Continuous rolling of a photo‐responsive LCN and its capability of climbing inclined surfaces. Reproduced with permission under the CC BY 4.0 license.^[^ ^91^ ^]^ Copyright 2016, The Authors, published by Springer Nature. b) Light‐controlled continuous rolling of a bilayer LCN/polypropylene actuator. Reproduced with permission.^[^ ^93^ ^]^ Copyright 2017, Wiley‐VCH.

In addition to manually changing the stimuli and triggering two (or several) different discrete actuation states, the actuation at a further level is to trigger continuous changes in actuation or trigger the deformation using computer‐aided microcontroller units. A noteworthy example by Wie et al. involved the illumination‐induced rolling of a twist nematic LCN strip (**Figure** **3**a). When exposed to a UV light, the LCN strip curled into a spiraled structure and continued to roll, even ascending slopes under illumination.^[^ ^91^, ^92^ ^]^ Practical considerations, including the size limitations of the illumination spot, necessitated the movement of the light source to track the rolling path of the spiraled structure.^[^ ^93^, ^94^ ^]^ In addition, Lu et al. demonstrated the ability to control the continuous rolling direction of LCPs by harnessing the pre‐stored strain energy within stretched LCPs. The ordering and relative positioning of light‐responsive LCPs in conjunction with intact transparent polypropylene influenced the rolling direction of the resulting circular wheels (Figure 3b).^[^ ^93^ ^]^ Similarly, for relatively large demonstrative wheels, the light source required adjustment during experiments to maintain focus on the moving sample.

**Figure 4:**
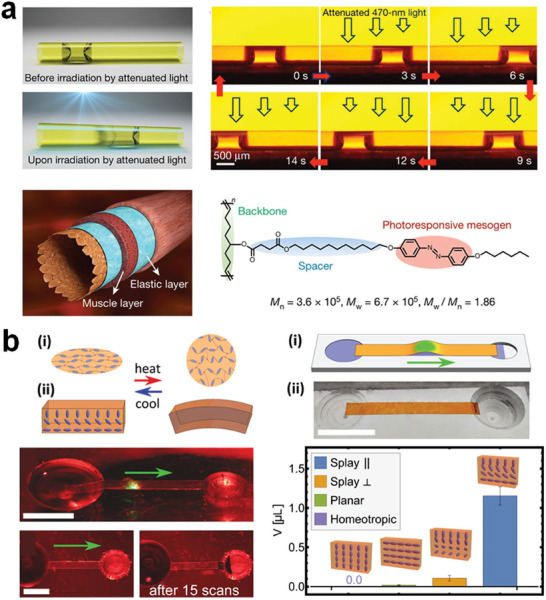
Continuous actuation for liquid transportation using light. a) Attenuated light‐powered motion of a liquid slug inside a microfluidic tube coated with photo‐responsive LCPs. Reproduced with permission.^[^ ^54^ ^]^ Copyright 2016, Springer Nature. b) Light‐driven peristaltic pumping by an LCN film of splay‐bend alignment. Reproduced with permission under the CC BY 4.0 license.^[^ ^96^ ^]^ Copyright 2023, The Authors, published by Springer Nature.

Beyond applications involving free‐standing robots, LCPs on supportive structures have found practical utility.^[^ ^54^, ^95^, ^96^ ^]^ For instance, Lv et al. developed LCP coatings within microfluidic tubes (**Figure** **4**a).^[^ ^54^ ^]^ By utilizing attenuated 470‐nm light to induce distinct shape changes within a small vicinity, they could control the movement of slugs by using actinic light. Similarly, a light‐powered peristaltic pump was demonstrated using a splay‐bend configuration of the LCN.^[^ ^96^ ^]^ By scanning the polymer stripe with actuating light, the LCN facilitated fluid movement between a film and a plate powered by a peristaltic deformation wave along the LCN strip (Figure 4b). These are two typical application examples of LCPs in closed and open‐surface microfluidics.

**Figure 5:**
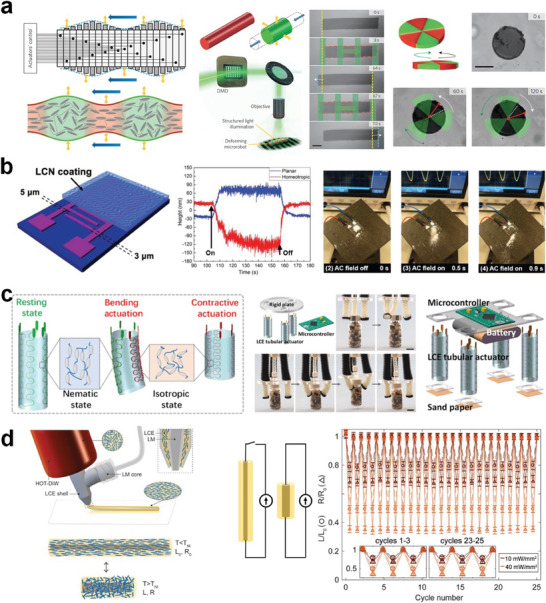
Control and actuation of LCPs using changing stimuli via computer‐aided methods. a) Actuation of the liquid crystal microrobot with structured light to generate traveling waves and robotic locomotion. Reproduced with permission.^[^ ^97^ ^]^ Copyright 2016, Springer Nature. b) Electrically responsive LCN coating with topographical deformation actuated by alternating electric fields and its application in water‐free remote cleaning. Reproduced with permission under the CC BY 4.0 license.^[^ ^32^ ^]^ Copyright 2018, The Authors, published by Wiley‐VCH. c) LCE robot with distributed and multimodal actuation via electrothermal effect and its application in the gripper and tubular actuator integrated with a microcontroller. Reproduced with permission under the CC BY 4.0 license.^[^ ^56^ ^]^ Copyright 2019, The Authors, published by AAAS. d) Innervated and self‐sensing LCE fibers are composed of a liquid metal conductive core and LCE shell. The LCE fibers are responsive to electrothermal actuation and have the capability for self‐sensing via the change in conductivity. Reproduced with permission.^[^ ^99^ ^]^ Copyright 2021, Wiley‐VCH.

The modulation of actuation signals can also be achieved through computer‐aided methods, with the potential for real‐time signal monitoring. For example, Palagi et al. employed a digital micromirror device to generate structured monochromatic light, inducing localized shape changes in illuminated regions. The resultant traveling deformation wave led to the propulsion of cylindrical microrobots and the in‐plane rotation of disc‐shaped microrobots (**Figure** **5**a).^[^ ^97^ ^]^ Apart from light‐based approaches, electricity offers an ideal means for remote control due to its ease of programming in terms of AC electrical frequency and strength or DC voltage facilitated by modern techniques. Consequently, electricity has been harnessed to control the deformation of LCPs, with the additional capability of sensing deformation and forming a “sensing, control, and actuation” closed‐loop control.^[^ ^32^, ^33^, ^56^, ^58^, ^83^, ^84^, ^98^, ^99^ ^]^ Feng et al., for instance, used alternating electric fields to create an oscillating LCN surface with a fingerprint molecular configuration. This electrically responsive coating provides an effective approach for remote cleaning of sand from photovoltaic devices without the need for water (Figure 5b).^[^ ^32^ ^]^ The oscillation of the coating surface was attributed to the oscillation of polar liquid crystal mesogens and the polymer network under the influence of the applied alternating electric field.^[^ ^100^, ^101^ ^]^ He et al. also developed tubular actuators employing electrothermal‐driven LCEs. With precise control using a microcontroller, they showcased distributed and multimodal actuation of multi‐armed/legged grippers and untethered robots (Figure 5c).^[^ ^56^ ^]^

In addition to actuation, sensing the actuation state is of paramount importance. The feedback signal can be optical property,^[^ ^49^ ^]^ or electrical resistance/conductivity.^[^ ^99^ ^]^ The change in conductivity induced by the shape deformation is usually manifested via conductive fillers (e.g., carbon nanotubes, graphene, ionic liquids, liquid metals) mixed in polymers or adhered conductive layers.^[^ ^88^, ^99^, ^102^, ^103^, ^104^, ^105^, ^106^ ^]^ For example, Kotikian et al. demonstrated electrical actuation and self‐sensing in conductive LCE–liquid metal composites.^[^ ^99^ ^]^ The composite comprised a liquid metal core encapsulated within an LCE shell, with the liquid metal serving dual purposes by facilitating electrical actuation and enabling self‐sensing through the monitoring of changes in conductivity during actuation (Figure 5d). This development holds significant potential, as the self‐sensing capability offers the prospect of creating autonomous soft robots when combined with microcontrollers and associated circuits.

In the above‐mentioned examples, various stimuli have been used to actuate the LC polymer actuators/robots. By examining studies chronologically, we can see that the research trend of stimuli‐responsive LCPs has gone through developing novel deformation modes and new stimuli‐responsive materials to application‐driven design of suitable responsive systems and devices for exact and practical scenarios. The application scenarios span various fields, ranging from self‐cleaning,^[^ ^32^ ^]^ microfluidics,^[^ ^54^, ^81^, ^107^, ^108^ ^]^ smart wearables^[^ ^109^ ^]^ to smart windows.^[^ ^110^, ^111^ ^]^ Toward other real and practical application scenarios, more functional monomers are desired and added to the LC library, and more alignment methods or structural designs of the actuators are expected to enrich the deformation modes of LC actuators.

## 3 Actuation with Static Stimuli

### 3.1 Oscillating Response Under Static Stimuli and Energy Input

**Figure 6:**
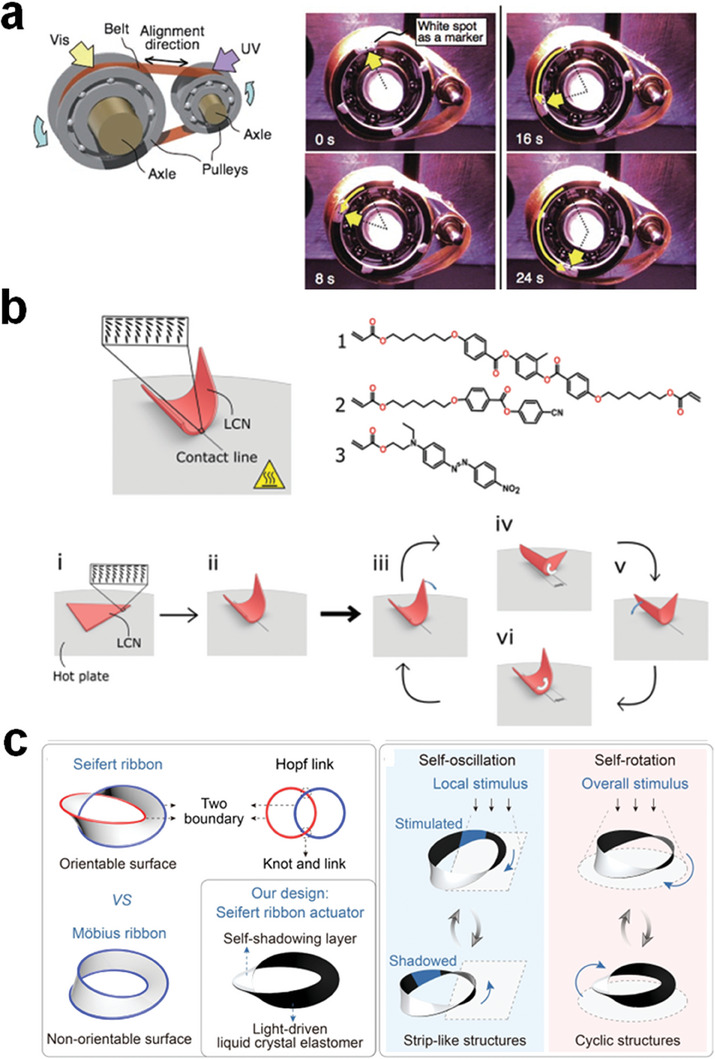
Self‐sustained actuation of LCP actuators. a) A plastic motor driven by light‐responsive LCE films. Reproduced with permission.^[^ ^112^ ^]^ Copyright 2008, Wiley‐VCH. b) Self‐sustained rocking motion of an LCN film with splay alignment and illustration of the underlying feedback loop. Reproduced with permission under the CC BY‐NC 3.0 license.^[^ ^113^ ^]^ Copyright 2019, The Authors, published by Royal Society of Chemistry (RSC). c) Continuous oscillation and rotatory motion of a LCE‐based Seifert ribbon. Reproduced with permission.^[^ ^116^ ^]^ Copyright 2023, Wiley‐VCH.

The characteristic of physical intelligence prominently manifests in scenarios involving static stimuli. A consistent stimulus field, such as a static heating or photonic field, engenders continuous actuation behavior (e.g., rolling, twisting, and bending) in physically intelligent devices. The self‐sustained actuation in a constant stimulation field contributes to the development of unmanned robotic systems. One form of continuous motion elicited within a static stimulus field is rolling. Yamada et al. engineered a rotary motor device employing a uniaxially aligned LCE belt wrapped around pulleys (**Figure** **6**a).^[^ ^112^ ^]^ The trans‐to‐cis isomerization of azobenzene mesogens generated local contractile forces, while visible light directed at the other pulley induced cis‐to‐trans isomerization, producing expansion forces. Similarly, open films can exhibit continuous rocking when exposed to static stimuli, such as heat and light.^[^ ^113^, ^114^, ^115^ ^]^ For instance, a triangular‐shaped LCN film with a splay alignment displayed self‐sustained rocking motion on a heated surface (Figure 6b), driven by the temperature gradient created above the heating stage.^[^ ^113^ ^]^ Moreover, the integration of light‐responsive mesogens also enabled the initiation of continuous motion through light exposure. Continuous motion was likewise observed in systems with more intricate geometries, such as topological ribbons. Nie et al. illustrated the photothermal effect‐driven continuous motion of a Seifert ribbon based on LCEs, with one side designed as a self‐shadowing platinum (Pt)‐coated passive layer (Figure 6c).^[^ ^116^ ^]^ The Seifert ribbon underwent oscillatory motion when partially exposed to light and rotational motion when fully illuminated.

**Figure 7:**
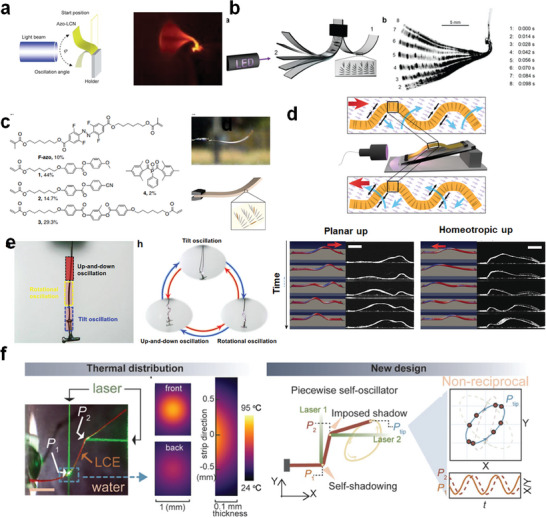
Oscillating actuation under static light stimulation. a) A cantilever azo‐LCN oscillator driven by a laser beam. Reproduced with permission.^[^ ^117^ ^]^ Copyright 2010, RSC. b) Self‐shadowing effect‐enabled mechanical oscillator with photothermal dyes. Reproduced with permission under the CC BY 4.0 license.^[^ ^30^ ^]^ Copyright 2017, The Authors, published by Wiley‐VCH. c) An intrinsic oscillatory LCN strip with splay alignment is achieved through the push–pull effect of the fluorine‐substituted azobenzene crosslinker. The isomerization reactions of the F‐azobenzene can be triggered by blue (405 nm) and green light (530 nm). Reproduced with permission under the CC BY 4.0 license.^[^ ^124^ ^]^ Copyright 2016, The Authors, published by Springer Nature. d) Wave propagation of the light‐fueled LCNs of splay alignment in a static light field. The propagation direction depends on the configuration of LCN placement. Reproduced with permission.^[^ ^3^ ^]^ Copyright 2017, Springer Nature. e) The photothermal effect and feedback loop induce multimodal oscillatory motions of an LCE fiber system. Reproduced with permission under the CC BY 4.0 license.^[^ ^125^ ^]^ Copyright 2021, The Authors, published by Springer Nature. f) Two orthogonal laser beams powered the non‐reciprocal oscillation of the LCE strip underwater. Reproduced with permission under the CC BY 4.0 license.^[^ ^121^ ^]^ Copyright 2023, The Authors, published by Wiley‐VCH.

Another class of actuators capable of continuous actuation within a static stimulus field, particularly a light field, are cantilever oscillators. As exemplified in **Figure** **7**a, a monodomain azobenzene LCN was actuated using a laser beam.^[^ ^117^ ^]^ The azo‐LCN demonstrated high‐frequency oscillations (reaching frequencies as high as 270 Hz). Notably, the cantilever exhibited greater amplitude when tested in a vacuum environment due to strong hydrodynamic loss in air. Employing a universal photothermal effect strategy, Broer's group demonstrated the oscillation of an LCN mechanical oscillator with splay alignment based on the self‐shadowing effect (Figure 7b).^[^ ^30^ ^]^ A hinge containing light‐absorbing dyes elevated the temperature upon light irradiation, leading to the bending of the splay LCN film, which, in turn, altered the illumination spot on the film, forming a feedback loop. It is worth noting that the self‐shadowing effect has been employed to develop multiple oscillators and adapted to other material systems, yielding intriguing applications such as phototropic pillars, oscillating cilia, phototactic swimmers, etc.^[^ ^70^, ^118^, ^119^, ^120^, ^121^, ^122^, ^123^ ^]^

Compared with the photochemical method via azobenzene isomerization demanding two light sources of different light wavelengths (Figure 6a), the self‐shadowing method only requires a static light field. Nevertheless, the self‐shadowing effect typically responds to high‐intensity light within specific angles, restricting its utility in confined spaces and unstructured environments. Consequently, there is a need to develop actuators with low actuation thresholds and independence from the direction of light.^[^ ^107^ ^]^

To create actuators that respond to low‐intensity light, such as sunlight, Zhao et al. combined modeling and designing materials with optimal elastic properties to develop a sunlight‐powered responsive LCE‐PDMS composite.^[^ ^70^ ^]^ This achievement was made possible by employing high‐photothermal efficiency fillers (candle soot), minimizing system damping, and selecting an LCE with a low transition temperature. To enable incident light angle‐independent self‐oscillation of free‐standing LCN beams or stripes, Kumar et al. employed a special azobenzene mesogen, F‐azo, which is responsive to 405 and 530 nm light, both of which are visible and part of the sunlight spectrum (Figure 7c).^[^ ^124^ ^]^ Consequently, F‐azo‐doped LCN exhibits oscillatory motions under sunlight. Despite its relatively small self‐oscillation amplitude (≈10° deflection angle), the underlying concept is very inspiring. Another effective strategy to induce self‐oscillation involves employing dual or complex light fields. For instance, Broer's group utilized two static light sources (at 455 and 365 nm) with alternating polarization directions to induce light‐powered topographical oscillations in LCN coatings.^[^ ^126^, ^127^ ^]^

The oscillatory motion of these LCP strips can be harnessed to develop various locomotive robots and functional devices. For instance, Gelebart et al. utilized the self‐shadowing effect to realize wave propagation in a fixed LCN strip placed on an inclined slope (Figure 7d), which proved effective in mitigating sand particle pollution.^[^ ^3^ ^]^ Framing the LCN film allowed the propagating wave to drive the locomotion of the soft walker. Under a constant light input, Hu et al. introduced complex motion into an LCE system, encompassing tilt oscillation, up‐and‐down oscillation, and rotational oscillation (Figure 7e).^[^ ^125^ ^]^ This LCE was further demonstrated to control the periodic motion of an attached reflector and modulate laser beams in a laser steering system. Beyond operation in air environments, the oscillation of the LCN system is also effective underwater, generating propulsive forces.^[^ ^81^, ^121^ ^]^ For example, based on the self‐shadowing effect, Deng et al. employed two orthogonal laser beams to create nonreciprocal strokes and pump fluids (Figure 7f).^[^ ^121^ ^]^ The nonreciprocal trajectory can be programmed by adjusting experimental parameters, such as the distance between the two laser spots.

Currently, most of the reported actuators in constant stimulation fields are powered by light. However, light is easily hindered by obstacles and not feasible to implement in confined spaces, limiting the development of LC polymer‐based unmanned robotic systems in these scenarios. Adoption of other energy systems, for example, electric field or alternating magnetic field, is a possible solution to developing unmanned robotic systems in an obstructed environment.^[^ ^128^ ^]^

### 3.2 Physical Intelligence Actuated by the Environmental Stimuli

The LCE/LCN robotic systems discussed above typically rely on deliberate energy inputs, such as light or electricity, to power their operations. However, a significant advancement in physical intelligence involves the development of robots that can harness energy from unstructured environments, perceive their surroundings, and make autonomous decisions. To work toward this objective, robots have been designed to respond to environmental stimuli, with elevated temperature as one of the most frequently employed environmental stimuli.

**Figure 8:**
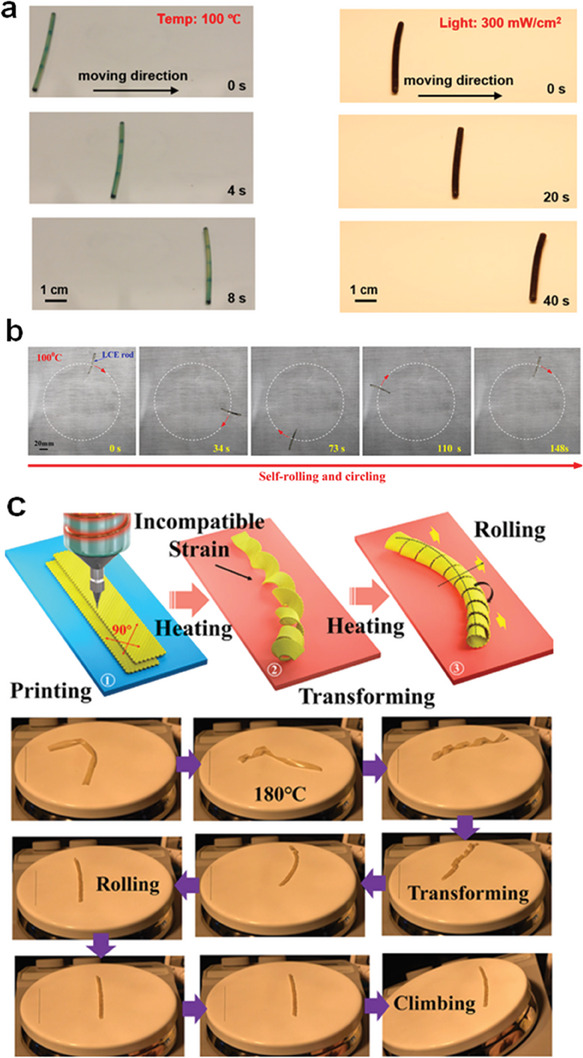
Heat‐sustained locomotion of LCPs. a) Sustained rolling of the LCE rod on a hot surface. Reproduced with permission.^[^ ^130^ ^]^ Copyright 2018, ACS. b) Self‐circling of a conical LCE rod on a hot surface. Reproduced with permission.^[^ ^134^ ^]^ Copyright 2023, Elsevier. c) Direct ink writing‐printed LCE rolls on the hot plate and its capability of climbing on an inclined surface. Reproduced with permission.^[^ ^135^ ^]^ Copyright 2021, Elsevier.

**Figure 9:**
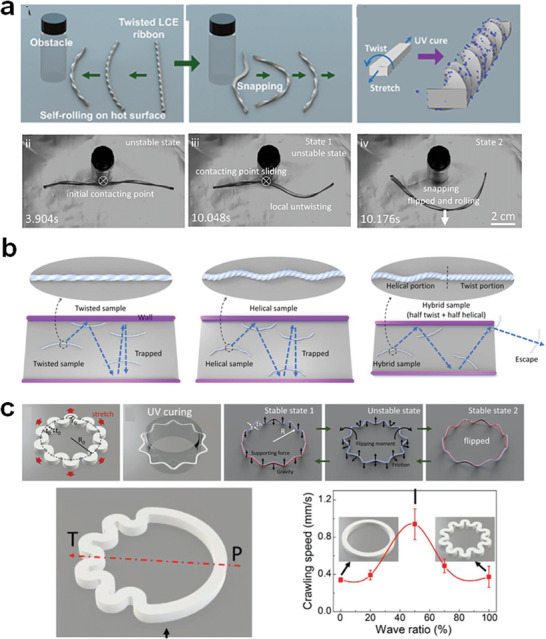
Heat‐sustained snapping and locomotion of LCPs a) Rolling and curvature snapping of the twisted LCE ribbon. Reproduced with permission under the CC BY‐NC‐ND 4.0 license.136 Copyright 2022, The Authors, published by Proceedings of the National Academy of Sciences (PNAS). b) The hybrid twisted‐helical LCE ribbon shows the improved capability of escaping the maze of two parallel walls. Reproduced with permission under the CC BY 4.0 license.131 Copyright 2023, The Authors, published AAAS. c) The snapping‐induced self‐flipping dancing motion of a freestanding LCE wavy ring and self‐crawling of asymmetric twisted wavy rings. Reproduced with permission.140 Copyright 2023, Wiley‐VCH.

The sustained motion of LCE robots induced by heat is typically achieved by creating a temperature gradient surrounding the heat source, such as a heating stage. This motion can oscillate around an equilibrium position^[^ ^113^, ^115^, ^129^ ^]^ or enable locomotion over significant distances.^[^ ^130^, ^131^, ^132^, ^133^ ^]^ In this section, our focus is on the latter, as we have previously discussed the former, and the expression of physical intelligence is more apparent during the locomotion process, akin to the evident intelligence in animals compared to static plants. Ahn et al. were pioneers in demonstrating the self‐sustained rolling of an LCE rod placed atop a hot stage (**Figure** **8**a).^[^ ^130^ ^]^ The rolling resulted from the non‐uniform temperature field along the elevated LCE rod, generating inhomogeneous actuation strain. The LCE rod continued rolling on the surface unless it encountered obstacles or another stimulus, such as interfering photothermal sources like light. When the uniform cylindrical rod shape was designed into a conical rod shape, the LCE exhibited self‐circling motion (Figure 8b).^[^ ^134^ ^]^ Liquid crystal alignment can also be configured into more complex patterns to create rolling robots. For example, Zhai et al. used direct ink writing to 3D print a bilayer LCE with orthogonal alignment directions (Figure 8c).^[^ ^135^ ^]^ The LCE sheet transformed into a helical structure and commenced rolling even on the heating stage, reversing its rolling direction when encountering obstacles. In parallel, Zhao et al. developed initially twisted LCE ribbons that could self‐roll on a heated stage (**Figure** **9**a).^[^ ^136^ ^]^ The rolling direction depended on the convex orientation of the curved centerline. It was irrespective of the twist's handedness, which is consistent with the theoretical model developed by Li, Cai, and coworkers.^[^ ^130^ ^]^ Interestingly, these twisted ribbons could also roll in dry, hot, loose sand and rocky terrains, snapping the convex orientation to reverse their rolling direction.^[^ ^137^, ^138^, ^139^ ^]^ Impressively, they possess the capacity to navigate mazes autonomously through spontaneous snapping and autonomous changes in rolling direction, ultimately escaping from the maze.

To enhance the adaptability of the rolling LCE and prevent entrapment between two parallel walls, Zhao and Yin et al. introduced asymmetry into their LCE ribbon model (Figure 9b).^[^ ^131^ ^]^ They developed a hybrid LCE ribbon composed of half‐twist and half‐helical shapes. While the LCE ribbon with only twisted shapes would oscillate back and forth between two parallel walls, the hybrid ribbons with both twisted and helical components exhibited asymmetric left‐ and right‐turning capabilities. This design endowed the LCE ribbon with the ability to navigate mazes, especially with parallel walls. From the above examples in Figure 8b and 9b,^[^ ^131^, ^134^ ^]^ we can see that introducing asymmetry/defect into the structural design of the robotic systems would sometimes introduce very interesting actuation, as also demonstrated in Figure 6c and other systems.^[^ ^116^, ^132^, ^140^, ^141^ ^]^

**Figure 10:**
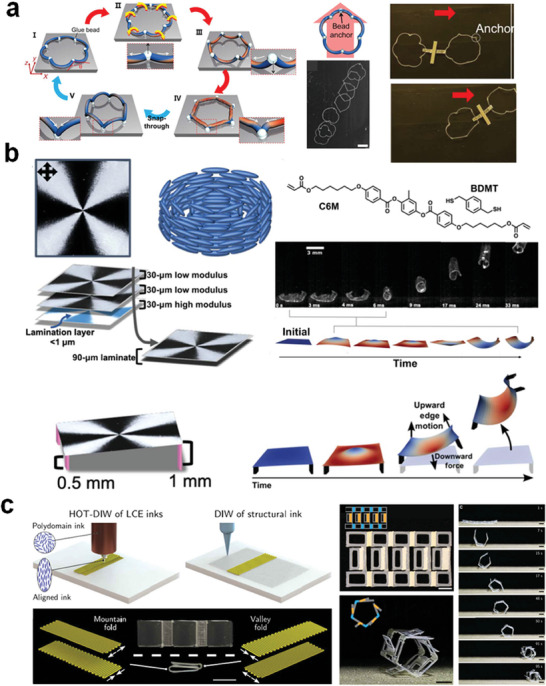
Heat‐induced locomotion of the LCE actuators. a) A rigid beads‐connected LCE loop with self‐sustained snapping, jumping, and propulsion on hot surfaces. Reproduced with permission under the CC BY 4.0 license.^[^ ^142^ ^]^ Copyright 2023, The Authors, published by AAAS. b) Leaping LCE sheet with modulus gradient along the thickness direction. The additional legs can be used to control directional leaping. Reproduced with permission under the CC BY 4.0 license.^[^ ^132^ ^]^ Copyright 2023, The Authors, published by AAAS. c) Rolling robot with LCE as the temperature‐responsive hinges. Reproduced with permission under the CC BY 4.0 license.^[^ ^133^ ^]^ Copyright 2019, The Authors, published by AAAS.

In addition to open‐loop ribbons, wavy ring‐shaped LCEs were developed to exhibit self‐sustained dancing motions (Figure 9c).^[^ ^140^ ^]^ Bistability was introduced during the molded fabrication process of the LCE ring, and the non‐uniform photothermal/heat field in the wavy ring induced self‐sustained snapping. The introduction of geometric asymmetry enabled directional lateral crawling of the wavy ring. Kim et al. developed LCE loops using rigid adhesive beads connecting multiple pre‐bent LCE fibers (**Figure** **10**a).^[^ ^142^ ^]^ This structural design, characterized by its instability and physical constraints, imparts the LCE loop with synchronized gait‐like motions. They demonstrated directional motions when introducing asymmetry into the LCE loop through a rigid anchor on the fiber. Furthermore, these LCE loops could function as legs for propelling the robot. In addition to 1D ribbon/stripe‐shaped LCE structures, LCP films have been engineered to exhibit snap and bistability.^[^ ^132^, ^143^, ^144^, ^145^, ^146^, ^147^, ^148^ ^]^ For instance, Hebner et al. created LCE sheets stacked in layers with a modulus gradient and +1 topological alignment (Figure 10b).^[^ ^132^ ^]^ These LCE sheets displayed rapid (6 ms) snapping responses and could leap to heights exceeding 200 times their thickness. By incorporating legs into the sheets, they could be engineered to perform directional leaps. The snapping reported by Hebner et al. is induced by intrinsic reversible deformation of the LCE sheet, therefore theoretically it can sustain many times which is different from a similar buckling PDMS‐hexane system relying on the evaporation of hexane solvent.^[^ ^139^ ^]^ The LCE can also act as a critical component to integrate into robots. Kotikian et al. employed direct ink writing to print LCE‐based hinges and utilized these temperature‐responsive hinges to develop self‐rolling robots on a hot surface (Figure 10c).^[^ ^133^ ^]^

### 3.3 Interactive Collective Robots Under Constant Stimuli

**Figure 11:**
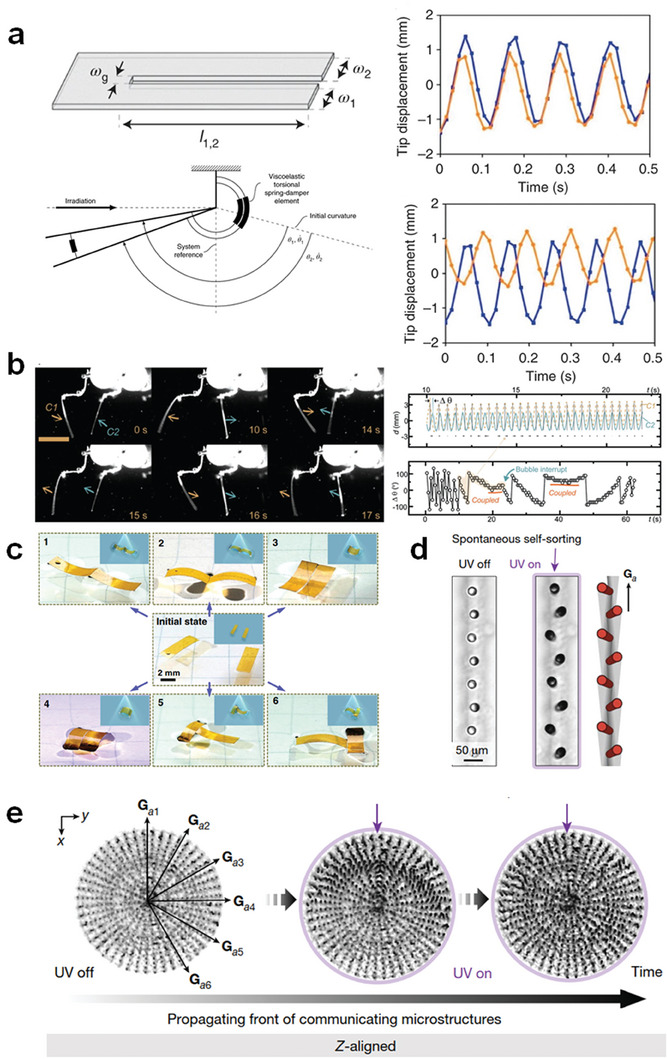
Collective LC polymeric actuators/robots. a) Collective synchronized oscillation of LCN films coupled via a joint LCN film. Reproduced with permission.^[^ ^122^ ^]^ Copyright 2021, Springer Nature. b) Oscillation of two LCE strips underwater coupled via the fluidic interaction. Two orthogonal laser beams actuated each LCE strip. Reproduced with permission under the CC BY 4.0 license.^[^ ^121^ ^]^ Copyright 2023, The Authors, published by Wiley‐VCH. c) Light‐programmed capillary assembly of LCN films. Reproduced with permission under the CC BY 4.0 license.^[^ ^161^ ^]^ Copyright 2020, The Authors, published by Springer Nature. d,e) LCN microposts pose self‐organization and traveling waves via light‐induced inter‐post “communication”. Reproduced with permission.^[^ ^4^ ^]^ Copyright 2022, Springer Nature.

In preceding sections, we have reviewed the progress of LCP‐based actuators and robots. The majority of these developments focused on individual units. However, nature often demonstrates the power of collective behavior among animals living in groups or swarms, such as ants, bees, and wild geese.^[^ ^149^, ^150^, ^151^ ^]^ At the microscopic level, the cilia on the surfaces of microorganisms coordinate their movements to propel fluids and nutrients.^[^ ^152^, ^153^ ^]^ The interactions between individuals lead to achievements surpassing any member's capabilities. To tackle complex tasks, robots have been developed to work in swarms, including magnetic swarms and drones.^[^ ^154^, ^155^, ^156^, ^157^, ^158^, ^159^, ^160^ ^]^ The emergence of robot swarms or groups constructed from LCPs is also gaining attention. For instance, Vantomme, Broer, and coworkers reported the collective synchronized motion of two LCN films.^[^ ^122^ ^]^ These LCN films were connected by joints made from the same LCN material. When actuated using photothermal and self‐shadowing effects, these coupled LCN films exhibited oscillations in either in‐phase or anti‐phase patterns (**Figure** **11**a). The specific synchronization mode depended on various environmental parameters, such as temperature and light intensity. Deng et al. demonstrated synchronized oscillations of LCE strips, actuated by two orthogonal laser beams underwater, by varying the coupling strength and oscillating frequencies of the two strips (Figure 11b).^[^ ^121^ ^]^ The difference between systems reported by Vantomme and Deng is that Deng's system requires two laser beams; but in both of two systems, the actuation is light‐beam angle‐dependent. Angle‐independent light‐powered systems would be desired as more synchronized actuators would be easily controlled in that case and the implemented instruments would not be too complicated for practical applications.

In addition to oscillations, researchers have explored other forms of synchrony. Hu et al. described the light‐programmed capillary assembly of LCN films.^[^ ^161^ ^]^ By controlling the magnitude and direction of attractive/repulsive capillary forces acting on these LCN films through light‐induced shape morphing, the authors could manipulate the assembly shapes and modes of these LCN films at the water surface (Figure 11c). Li, Aizenberg, and their collaborators employed LCN micropillars and microposts, where the LC director did not align with the principal or geometrical axes, to exhibit various non‐reciprocal motions.^[^ ^4^, ^36^ ^]^ The arranged microposts “communicated” with one another through the shadowing effect during light illumination, resulting in spontaneous self‐organization and collective traveling waves (Figure 11d,e).^[^ ^4^ ^]^ Despite recent impressive progress in the collective actuation of LCP actuators/robots, there is great potential in developing such systems using LCP actuators with increased system complexity, reconfigurability, and functionality when compared with robot swarms made from other materials, for example, magnetic microrobots.^[^ ^154^, ^160^, ^162^, ^163^ ^]^

### 3.4 LCPs as Sensors Integrated into the Robotic Systems

Instead of actuation directly triggered by external stimuli, LCEs can be integrated into soft robots as sensors, building the connection between the robotic systems and their surrounding environments. Compared to conventional mechatronics‐based robotic systems to achieve autonomy, this distributed strategy offers unique advantages such as simplicity and scalability. For instance, He et al. demonstrate the autonomous path‐planning capability based on embodied and distributed physical intelligence.^[^ ^164^ ^]^ Specifically, LCE strips are distributed to a kirigami‐inspired pneumatic robot. The robot moves straight without external stimuli. When subjected to light or heat, the embodied LCEs contract locally constrains the kirigami. As a result, the shape of the robotic body automatically changes, causing the robot to steer close to the light or heat autonomously. Multiple moving strategies, such as steering away from and moving toward stimuli, have been demonstrated by adding, removing, or rearranging the LCE strips. In addition to the trajectories’ change, the locomotion of the robotic systems may also be switched using the same strategy. Zhang et al. demonstrated such behavior by developing a self‐adaptable LCE‐based magnetic robot.^[^ ^82^ ^]^ Driven by the external magnetic field, this robot moves in a cool, dry substrate following a walking gait. Once the robot is placed in a viscous liquid with high temperature, the robotic body transforms from a flat sheet to a helix configuration. As a result, this machine autonomously changes to a swimming mode, which is a more efficient approach for locomotion inside liquids. Similarly, Sun et al. developed a programmable magnetic‐LCE strip robot via additive manufacturing.^[^ ^165^ ^]^ This robot exhibits versatile actuation modes under complex environments, including crawling, rolling, twirling, and self‐propelling, showing excellent adaptability. These abovementioned examples provide new opportunities to construct next‐generation autonomous robots with unique features of simplicity and scalability, using LCPs as sensors. However, to develop functional sensors for other practical applications, corresponding liquid crystalline monomers with specific responsive chemical groups remain to be designed and synthesized according to the characteristics of the environment, for example, specific monomers in response to biochemical signals for biomedical applications.

## 4 Robotic LC Polymer Systems Capable of Learning and Logic Control

**Figure 12:**
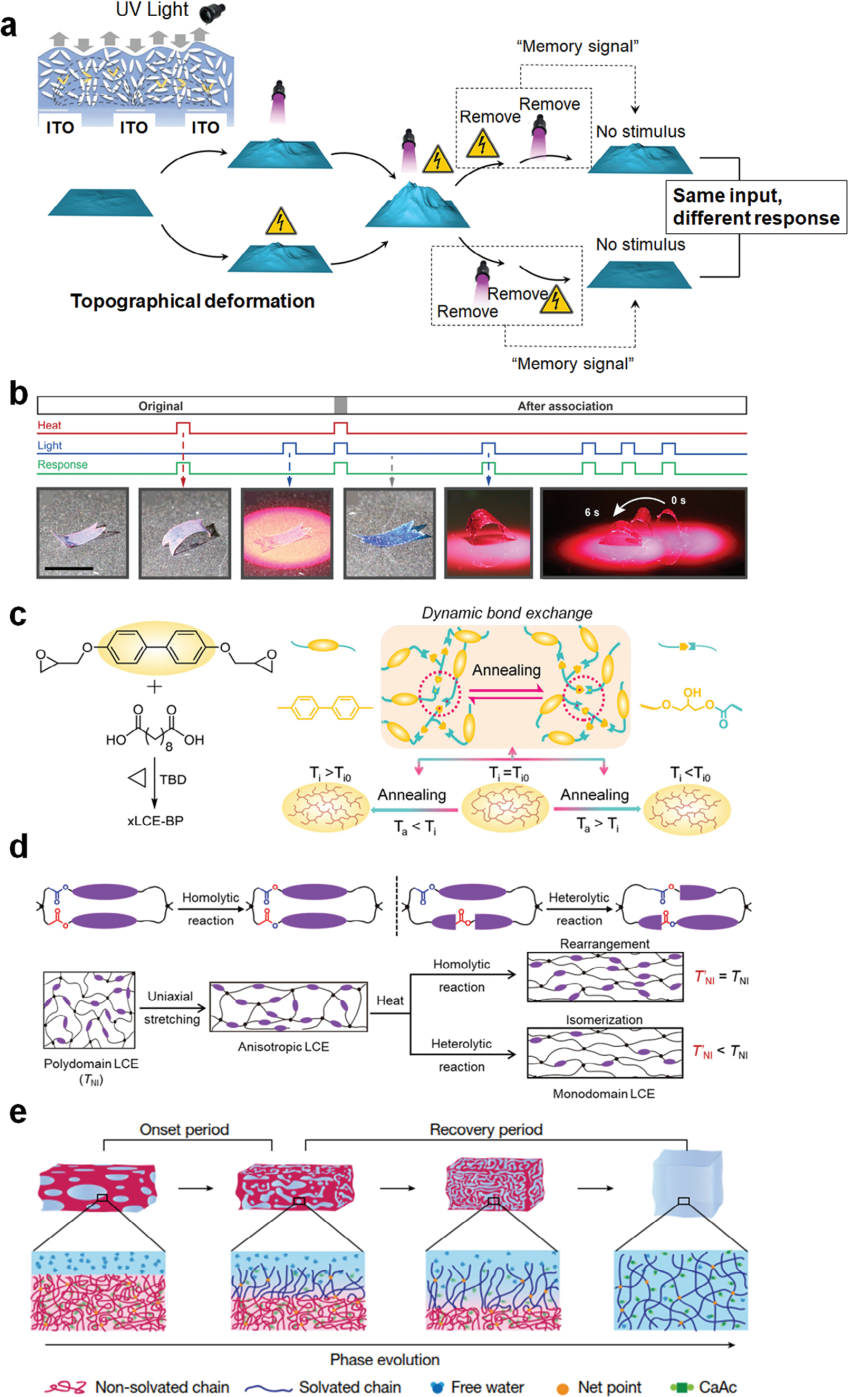
Robotic systems capable of learning and memorizing. a) LCN coating with sequential logic control using combined electric field and light stimuli. Reproduced with permission.^[^ ^33^ ^]^ Copyright 2020, Wiley‐VCH. b) Associated learning of the LCN in response to heat and light stimuli. Reproduced with permission under the CC BY‐NC‐ND license.^[^ ^168^ ^]^ Copyright 2020, The Authors, published by Elsevier. c) Tunable actuation temperature for LCEs by annealing the LCEs with covalently adaptable networks. Reproduced with permission under the CC BY 4.0 license.^[^ ^169^ ^]^ Copyright 2023, The Authors, published by Springer Nature. d) Programmable actuation temperature of LCEs via isomerization of the polymer network topology. Reproduced with permission under the CC BY 4.0 license.^[^ ^170^ ^]^ Copyright 2023, The Authors, published by Springer Nature. (e) Programmed actuation of shape memory hydrogels assisted by time‐dependent phase separation. Reproduced with permission.^[^ ^166^ ^]^ Copyright 2023, Springer Nature.

In addition to the physical adaptation to environmental stimuli, the learning ability represents a new dimension of intelligence. Compared to machine learning, which relies on digital/virtual algorithms, one critical part aspect of physical learning lies in materials development. The relevant research about associated learning and logic control of robots is still in its infancy with limited examples. One of the key points in designing such systems is to introduce “memory” into the system. The “memory” can be encoded into materials via chemical reaction hysteresis (e.g., isomerization of azobenzene),^[^ ^33^ ^]^ time‐dependent material diffusion (e.g., solvent or other substances),^[^ ^166^, ^167^ ^]^ or topology change of polymer chains/network. For the hysteresis strategy, Feng et al. showcased the sequential logic control of surface topographical deformations in LCN coatings in response to electrical and light stimuli (**Figure** **12**a).^[^ ^33^ ^]^ The cumulative topographical deformation resulted from the combination of electrical and light stimuli, with the final state contingent on the sequence of deactivating the light and electric field. This was made possible through electromechanical, electrothermal effects, and the thermally accelerated cis–trans isomerization of azobenzene groups.

Time‐dependent material diffusion (e.g., solvent or other substances) is an alternative strategy to develop smart systems capable of learning or logical control. Zeng et al. introduced the concept of associative learning in LCN actuators by simultaneously employing heating, photothermal effects, and dye diffusion (Figure 12b).^[^ ^168^ ^]^ When subjected to both heating and light irradiation, the dye, with its photothermal effect, diffused into the LCN, enabling the LCN to exhibit new photothermal‐induced bending behavior, referred to as associative learning. Notably, this material diffusion strategy is not limited to LCNs but has also been demonstrated in other material systems, for example, hydrogels, to exhibit programmed shape memory performance and alternation of mechanical strength.^[^ ^166^, ^167^, ^171^ ^]^

Another alternative feasible strategy is to alter the topology of the LCE polymer network. Recent examples include changing the actuation behavior/temperature of LCEs via changing the polymer network topology or structural order (Figure 12c,d).^[^ ^169^, ^170^ ^]^ By changing the network structure in certain situations (e.g., annealing at a certain temperature), the actuation performance of actuators can be changed by the annealing “experience”.

## 5 Conclusion and Outlook

Physical intelligence has garnered significant attention in recent years, and research involving LCPs has made substantial advancements owing to their remarkable reversible shape‐morphing capabilities in response to external stimuli. The progression of this research has evolved from manual “two‐state” actuation to digitally engineered continuous oscillation and, more recently, to self‐regulated multimodal actuation with logic and learning capabilities. Scientifically, more efforts are needed to develop high‐performance smart devices and robot swarms based on LCPs. Most of the developed actuators based on LCP still could not match or exceed the performance of their natural counterparts‐human arms and muscles not only in energy output but also in programmability and degree‐of‐freedom, despite a few examples showing impressive power density.^[^ ^172^ ^]^ Moreover, the study of physical intelligence is still in an early stage: the deformation mode of a certain LCP is determined once it is fabricated, and learning capability to perform new deformation modes is still not possible. At the swarm level, some underlying mechanism of the collective motion of LCPs remains vague and the developed devices are also in a preliminary stage. To develop an intelligent swarm using shape‐morphable LCPs, learning from nature is perhaps a possible approach. For example, the aerodynamics and interaction between individuals of the flock of wild geese can be learned and adapted to develop flying LCPs using flapping wings. Developing flying LCPs without the aid of wind tunnel, however, will certainly be the first step which in turn commands for energy‐efficient electrically or other energy‐powered LCPs as wings.^[^ ^173^, ^174^, ^175^ ^]^

In addition, several technical challenges still need to be addressed before practical applications can be realized.

### 5.1 Power Density

Actuators and robots based on LCPs exhibit versatile deformation modes. However, to perform practical work, a requisite force output is necessary. One of the most important of actuation performance for evaluation is power density. The power density (*P* = *εσf*) is proportional to strain, stress, and frequency. The power density for LCE‐based actuators is typically low (one order of magnitude lower than human muscle), limiting practical applications. Unfortunately, the output force of LCNs/LCEs remains constrained when keeping the response speed at an acceptable level, despite some studies aimed at amplifying it.^[^ ^176^, ^177^ ^]^ While other material systems can provide substantial force output, such as HASEL (hydraulically amplified self‐healing electrostatic) actuators,^[^ ^178^ ^]^ combustion actuators^[^ ^179^ ^]^ or Nylon,^[^ ^180^ ^]^ they often involve high driving voltages unsuitable for practical applications or require tethering. Enhancing the power density of LCPs would significantly enhance their practical applicability.

### 5.2 Types of Stimuli

Most reported LC polymer actuators rely on manually arranged stimuli in structured conditions, for instance, heat (photothermal/electrothermal/magnetothermal), light, humidity, solvents, electricity, and pressure.^[^ ^7^, ^30^, ^51^, ^52^, ^67^, ^87^, ^131^, ^181^, ^182^, ^183^, ^184^ ^]^ To develop autonomous robots, especially in environments where light is obstructed, or underwater conditions where electronic components are inconvenient to employ, and heat dissipation is high, new stimuli tailored for LCPs and corresponding LC monomers for their fabrication remain largely unexplored. Insights from chemotactic particle microrobots could potentially inform the exploration of new stimuli for LCPs.^[^ ^185^, ^186^ ^]^

### 5.3 Microfabrication Methods

The distinctive characteristic of stimuli‐responsive LCPs over conventional mechatronics‐based systems is that they behave with physical intelligence, meaning they spontaneously sense the environment and act by self‐decision. This will pose a great advantage for LCPs on a small scale and in confined spaces. However, current methodologies for fabricating small‐scale LCP actuators and robots are relatively limited, primarily encompassing techniques such as laser cutting and two‐photon polymerization for devices requiring manual assembly.^[^ ^4^, ^78^ ^]^ The development of efficient methods for fabricating miniature LCP actuators with diverse actuation modes remains an intriguing challenge.

By incorporating physical intelligence into actuators and robots, LCPs offer substantial potential for creating “soft” intelligent devices that can operate without relying on traditional “rigid” electronic sensors. We hope that the progress and challenges outlined in this review will inspire the development of smarter actuators and robots with physical intelligence, not only utilizing LCPs but also exploring the possibilities with other materials.

## Conflict of Interest

The authors declare no conflict of interest.
